# Using Digital Tools for Contact Tracing to Improve COVID-19 Safety in Schools: Qualitative Study Exploring Views and Experiences Among School Staff

**DOI:** 10.2196/36412

**Published:** 2022-11-01

**Authors:** Sofia Chantziara, Amberly Brigden L C, Claire H Mccallum, Ian J Craddock

**Affiliations:** 1 Faculty of Engineering University of Bristol Bristol United Kingdom

**Keywords:** schools, contact tracing, COVID-19 mitigation, COVID-19, pandemic, disease prevention, health technology, COVID-19 management, technology support, digital tool, mobile health, mobile technology

## Abstract

**Background:**

Throughout the pandemic, governments worldwide have issued guidelines to manage the spread and impact of COVID-19 in schools, including measures around social distancing and contact tracing. Whether schools required support to implement these guidelines has not yet been explored in depth. Despite the development of a range of technologies to tackle COVID-19, such as contact-tracing apps and electronic vaccine certificates, research on their usefulness in school settings has been limited.

**Objective:**

The aim of the study was to explore the needs of school staff in managing COVID-19 and their experiences and perspectives on technological support in relation to contact tracing. School staff are the ones likely to make key implementation decisions regarding new technologies, and they are also the ones responsible for using the new tools daily. Including both management staff and class teachers in the development of school-based technologies can lead to their successful adoption by schools.

**Methods:**

Semistructured interviews were conducted with UK school staff, including primary and secondary school teachers and school managers. Thematic analysis, facilitated by NVivo, was used to analyze the data. Two of the authors independently coded 5 (28%) of the interviews and reached a consensus on a coding framework.

**Results:**

Via purposive sampling, we recruited 18 participants from 5 schools. Findings showed that primary schools did not perform contact tracing, while in secondary schools, digital seating plans were used to identify close contacts in the classroom and manual investigations were also conducted identify social contacts. Participants reported that despite their efforts, high-risk interactions between students were not adequately monitored. There was a need to improve accuracy when identifying close contacts in common areas where students congregate. Proximity tracking, use of access cards, and closed-circuit television (CCTV) emerged as potential solutions, but there were concerns surrounding false alerts, burden, and security.

**Conclusions:**

School staff have found it difficult to monitor and implement social distancing and contact-tracing provisions. There are opportunities for mobile digital technologies and CCTV to support school staff in keeping their students and colleagues safe; however, these must place minimal demands on staff and prioritize security measures. Study findings can help researchers and practitioners who work in different contexts and settings understand what particular challenges are faced by school staff, and inform further research on the design and application of digital solutions for contact tracing.

## Introduction

### Background

Many governments worldwide used school closures as a way to temporarily reduce the spread of COVID-19. In the United Kingdom, schools closed the first time between March 18 and June 1, 2020, and a second time between January 4 and March 8, 2021 [1]. Since March 2021, schools have remained open; however, in the context of increased community transmission, high rates of COVID-19 have been observed among school-age children [2,3]. There are also strong indications that older children (10-16 years) can facilitate transmission and are more likely to introduce infections into a household compared to adults [3]. Previous research has suggested that there is a greater possibility for larger disease outbreaks in secondary schools compared to primary schools [4,5].

School closures should be avoided as they have a negative impact on children’s social, physical, educational, and psychological development, with students from lower-income backgrounds impacted disproportionately [6]. They also affect parents’ ability to work, (particularly women’s), resulting in lower productivity and loss of income [6,7]. Supporting schools to stay open is crucial, yet it requires systems, such as testing, contact tracing, social distancing, and other support, to mitigate against infection transmission and to give students, staff, and parents confidence [8].

Over the course of the COVID-19 pandemic, countries have issued various sets of guidelines for schools [9-11]. These included measures for social distancing (eg, maintain distinct groups of pupils, minimize contact across the school site, stagger school start and finish times, maintain distance between teachers and pupils and between teachers) along with the use of regular testing, face coverings, contact tracing, and quarantine/testing of close contacts. Guidelines usually leave a certain degree of flexibility to schools to implement these measures in a way that meets the needs and age range of their students, the physical layout of each school, and the resources available [9]. School staff have nevertheless voiced concerns about how the guidelines can be followed in practice [12,13]. Since the implementation of these measures, little has been published on how school staff were managing and any challenges they have faced.

Over the past 2 years, technologies have been proposed to support COVID-19 management in several ways, including monitoring, surveillance, detection, and prevention [14,15]. One of the most high-profile uses of technology in tackling COVID-19 has been for contact tracing—a key measure in preventing the spread of infectious diseases [16]. Contact tracing involves identifying people who have been in contact with an infected individual and their subsequent isolation [16]. Digital technologies that support contact tracing, such as GPS chips capable of precise location tracking, Bluetooth radios that can sense the proximity between devices, and always-on connections to the internet, can increase efficiency over more labor-intensive manual methods [17,18]. Private companies have developed systems with the aim to assist schools and other businesses with contact tracing [19,20]; however, no studies so far have assessed their use for contact tracing within schools. A number of studies have aimed to investigate transmission models in schools and found that wireless sensors can more accurately identify close-proximity contacts of short durations compared to self-reported measures [21,22]. However, these studies were conducted before the COVID-19 pandemic and they did not look into the real-world adoption of these systems by schools.

Although contact-tracing tools have not been widely implemented in schools during the pandemic, surveillance technologies are already prevalent in UK, US, and Australian schools [23-25]. Biometric technologies, such as fingerprint scanners, have been used for library management and cashless catering [26,27]. Systems equipped with facial recognition technology, although less prevalent, have been installed in schools with the aim of detecting sex offenders or finding missing children [28] or to facilitate canteen payments [29]. The use of closed-circuit television (CCTV) is commonplace in many UK schools, and it is used primarily for the purpose of crime prevention and detection [24]. Previous studies on the acceptance of CCTV within schools have shown that perceived invasion of privacy relies upon a number of factors, such as the location of the cameras, their rationale, and whether individuals are being monitored continuously [26]. Studies have also suggested that a balance could be struck between the use of CCTV and the impact upon privacy [30].

Recent studies have highlighted that willingness to use contact-tracing technologies can be negatively affected by privacy concerns [31,32], even when concerns about COVID-19 remain equally high [31]. The use of these technologies raised concerns as individuals believed that contact tracing will involve increased surveillance by the government [30], were worried about third parties accessing their personal data, and had misunderstandings regarding what contact tracing will entail [30]. There are also examples of digital health programs that were abandoned as they failed to win public trust because of fears over privacy breaches and protection of anonymity [33,34].

It is important that any tools developed for schools be co-designed with potential users and stakeholders since this process ensures that they are more likely to be usable and engaging [35]. The first step in co-design is understanding the views and needs of potential users [36-38]. Exploring the context in which the tools would be deployed, and the views of potential users, can help ensure that any technology developed is likely to be useful, engaging, acceptable, and feasible to implement [39,40]. School staff, including both management staff and class teachers, are a key user group to involve in the development of school-based technologies [41,42]. The views of school staff can play a pivotal role in whether technologies in schools are successfully adopted [43,44], and they are also likely to make key implementation decisions and be responsible for using the tools daily.

### Objective

In this study, we explored the challenges experienced by school staff in their efforts to limit the spread COVID-19 within the school, opportunities for technologies to support contact tracing, and considerations for the design of such technologies. The study was part of the wider COVID-19 Mapping and Mitigation in Schools (CoMMinS; R101587-103), a National Institute of Health Research (NIHR), UK Research and Innovation (UKRI) project, which aimed to iterate and evaluate COVID-19 control and mitigation measures in schools through a program of active and responsive research conducted in partnership with schools.

## Methods

### Recruitment

Data for this study were collected from a subsample of primary and secondary schools in the wider area of Bristol (UK) that participated in the wider CoMMinS project. Special education needs schools were not included. We aimed to recruit a diverse range of schools based on the percentage of students from Black, Asian, and minority ethnic (BAME) groups; the percentage of students receiving funding to improve educational outcomes (student premium); the percentage of students eligible for free school meals (FSM); and indices of multiple deprivation (IMD). These data were ascertained from the UK government website [45]. Schools were also selected based on their capacity and willingness to engage in the study.

Participants were eligible if they were staff who held a teaching role, IT role, or school management role (eg, heads and deputy heads) or were otherwise tasked with managing COVID-19 within the school. Participants also needed to have access to video-call facility and be able to speak English. To recruit staff, a key contact was identified within each school, who liaised with the first author to advertise the study to the members of staff. This process included circulating an invitation letter to staff members, which included a link to the participant information sheet. In addition, a link to an online expression-of-interest form (via the electronic system REDCap [Vanderbilt University], a secure online data capture system designed exclusively for research [46]) was provided. On this expression-of-interest form, the participants were asked to provide contact details and were asked to provide consent for a researcher to contact them to provide full information about the study.

### Ethical Considerations

Individuals who registered their interest in REDCap were contacted by the first author, who ensured that they were provided with and understood all the relevant information about the study. Informed consent in writing was obtained through REDCap from those individuals who agreed to participate. Participants were offered a £20 (US $22.18) voucher as a thank-you for taking part. All procedures were approved by the Faculty of Life Sciences Research Ethics Committee at the University of Bristol (reference no. 112284).

### Data Collection

This qualitative study was grounded in the theory of phenomenology. Phenomenology aims to understand the meaning, structure, and essence of a lived experience of a particular phenomenon for individuals or a group of people [47]. In this context, the primary aim of qualitative research is to develop an understanding of how the world is constructed by the individuals involved in the research situation [48]. The aim of this study was to provide a deep understanding of participants’ experience of the challenges they faced in their efforts to limit the spread COVID-19 within the school and their views on technologies that can support contact tracing.

Semistructured interviews were conducted between February and July 2021 by the first author via video calls. One of the benefits of interviews is the richness of data they can produce [49] compared to focus groups, which are more likely to give rise to attitudes, opinions, and third-person stories [50]. Semistructured interviews were chosen over fully structured ones as they promote a dialogue and allow the interviewer to explore in depth the thoughts, views, and experiences of participants, while additional questions can elicit more detailed narratives and stories [51]. As this study focused on an underinvestigated area, the aim was not to explore predetermined theories or themes and it was considered important to allow the participants during the interviews enough space to share their experiences and views.

A topic guide was developed by the research team to cover the main aims of the study. In the context of semistructured interviews, the topic guide was developed iteratively and refined and adjusted as interviews progressed (Textbox 1). The last section of the topic guide in particular (ie, digital solutions presented for discussion) was updated from one interview to the next as digital solutions were either suggested by participants (digital proforma, proximity tracking) or proposed by the researchers (CCTV, access cards, digital seating plans) based on participants’ descriptions of the challenges they were facing when they were trying to identify close contacts of positive cases. During the first 2 interviews, no solutions were included in the topic guide. As the research team developed an understanding of how schools proceed with contact tracing and what were the blind spots in this process, they started formulating suggestions about digital tools that could be used to increase accuracy. Participants were also asked to provide their own ideas about digital tools. As interviews progressed, tools suggested by participants and researchers were added to the topic guide. Interviews lasted, on average, 30 minutes and were audio-recorded with participants’ consent.

**Topic guide.**

**Participant/school characteristics**

- What is your job role, and what does that involve in terms of managing COVID-19?
- Which areas, if any, can be characterized as hot spots for transmission (ie, areas where students/staff could interact)? How adequate are provisions for social distancing?

**Current adherence to government guidance**

- Can you tell us about the school’s procedures for the management of COVID-19 in terms of social distancing and contact tracing?
- What happens if there is a positive case? What information is collected on the case?
- How do you identify contacts with the case? What information is used to identify contacts?
- Whom does the school notify (families, local authority), or who is alerted to the case?
- How do you record/evidence conversations?
- Does the school need to follow up/check-in on the case?
- Are there any steps to protect the anonymity of the case?

**School staff needs and barriers to adherence**

- What parts of the process are challenging?
- Do any parts/processes not work? If so, why not?
- What support, if any, do you/the school need to adhere to/implement guidelines?

**Digital solutions**

What technologies, if any, might help to support you/the school in managing COVID-19?

- Does your school have closed-circuit television (CCTV) and in which areas?
- Who has access to the footage? Do you need to get approval to review footage?
- What are your thoughts on using CCTV footage to identify close contacts?
- Do you think this could feasibly be reviewed to look at contact tracing outside the classroom (eg, lunch hall)?
- Do you foresee any challenges?

What are your thoughts on using proximity tracking for contact tracing?

Do you foresee any challenges? What are your thoughts on using access cards to enter common school areas? Do you foresee any challenges?

What are your thoughts on digital seating plans?

- Are pupils sticking to the seating plan? Are there are circumstances where they move around the classroom or change their desk?
- Primary schools: Do you have a seating plan, or are children mixing a lot within the class (may differ depending on the primary year group)?
- Are pupils allowed to have phones in schools, and are they allowed to use them?
- What are your thoughts on quick response (QR) codes on seats/tables to update seating plans?

What are your thoughts on a digital proforma that could be accessed and completed by the school and authorities?

- Would this make it easier/quicker for the school to collect and report information?
- Do you foresee any problems?

### Analysis

Interviews were transcribed verbatim, and transcripts were pseudonymized. Thematic analysis was used to analyze the data, following the principles of a 6-stage process, as outlined by Braun and Clarke [52]. Analysis was facilitated by NVivo 12 (QSR International) [53]. All themes were produced inductively and were linked closely to participants’ accounts. Two of the authors independently coded 5 (28%) of the interviews. The authors reached a consensus on a coding framework, and any disagreements were resolved through discussions before refining and finalizing themes and subthemes.

## Results

### Participants

The study included 5 schools: 1 primary (age range of students 5-11 years) and 4 secondary (age range of students 12-18 years). For 3 (60%) of the 5 schools, diversity measures were available. The sample included schools with very high to very low deprivation scores, including 1 independent school. There was also diversity regarding the percentage of BAME students (38%-72%), students eligible for FSM (12%-36%), and students receiving a pupil premium (33%-56%). We initially aimed to purposively sample from the pool of interested participants, aiming for diversity on the role in school. However, because of the constraints in the number of participants expressing interest, recruitment was opportunistic, and we sampled all participants who completed an expression-of-interest form. All individuals (N=42) who provided their contact details via REDCap were contacted by the first author. Those who responded and agreed to participate were included in the study. Data collection ended when the research team concluded that any new information would have a minor or no influence on themes that already were emerging from participants’ accounts and it was believed that saturation was reached [54]. Across the 6 schools, 18 (42.9%) participants were recruited. Of the 18 participants, 8 (44.4%) were female and 10 (55.6%) male; in addition, 4 (22.2%) were senior management, 12 (66.7%) were teachers, 1 (5.6%) a teaching assistant, and 1 (5.6%) a behavior support manager.

### Themes

Three themes were identified that described the school staff’s efforts and challenges in managing social distancing and contact tracing and their suggestions for digital solutions that could enhance existing provisions. These themes and subthemes along with quotes from interviews are outlined in Table 1 and described in detail later.

**Table 1:** Themes and subthemes.

Themes and subthemes		Interview quotes
**Social distancing measures are in place to prevent and limit high-risk interactions; however, blind spots exist.**		
	Classrooms are a relatively controlled environment, with strict and easily observed social distancing measures in place in secondary schools, while more flexibility is allowed in primary schools.	“The children, they were given seating plans, and the children had to stay in their seating plan, the teacher didn’t mingle in with the pupils, so that...that was easy to manage inside the classroom.” [Teacher S^a^] “In early years and key stage 1, we do have group worktables, but they are a meter apart…the tables, so they’re one either side. Or if they’re next to each other, they’re side by side rather than directly facing each other.” [Teacher P^b^]
	Mitigation measures are in place to prevent mixing between “bubbles”/year groups, and although social distancing is encouraged, it is not always possible.	“We were able to stagger when different year groups were leaving school, entering school, and the different year groups had different areas in the school.” [Teacher S] “As soon as they all come out of those classrooms, you know, so there will always be excessive close contact in a corridor. I don’t think logistically...that’s very avoidable.” [Teacher S]
	Despite the existing measures, potentially risky interactions are still taking place, and mobile systems that monitor proximity between teachers and students could increase adherence.	“Also, with teaching, it’s effectively impossible to teach from the front of the class at all times, so there are certain times, um, that we find it a real struggle within our subjects.” [Teacher S] “A device that alerts you to the fact that you have been with this person or close to this person for more than 10 minutes now, it would be helpful.” [Teacher S]
**School** **staff use both manual and digital methods to support contact tracing; however, greater accuracy is needed.**		
	Contact tracing in secondary schools includes reviewing seating plans and consulting with the positive case and their circle of friends, staff, and family, and in this process, protecting the anonymity of the case is not the main priority.	“Each case we had, we asked them were they happy with us sharing the information that they had tested positive so that we could trace any contacts, and every time they said yes, so with the pupils, there’s not really any way around it.” [Teacher S] “We speak to the positive student, and if that means a phone call, that’s a phone call home, and we just say, ‘Who are your close contacts? Who are your friends? Who do you have lunch with?,’ and then they tell us, and then, from there, we contact those students that have been named.” [Teacher S]
	In primary schools, contact tracing does not involve elaborate investigations, and in the presence of a positive case, whole bubbles are required to isolate.	“[We] can’t rely on them (the students) at all, and even, even with year 7 and 8, it’s, it’s difficult, because again I think the stigma around being isolated at the time meant they wouldn’t want to tell you who they’d been in contact with.” [Assistant head P and S] “If they’d all been in their classes, no problem, you could just send one class home. If they’d been in mixed maths classes or a mixed PE^c^ class, then we could have ended up in the situation where we would have to send potentially an entire year group home, depending on what they’d been doing, or at least a combination of bubbles might have to go.” [Assistant head P and S]
	Despite the manual investigations, students’ cooperation, and the use of existing digital systems, contact tracing is not always comprehensive and accurate.	“We had lots of students that wanted to be involved in the first case. You know, it was exciting they wanted to be a close contact. They thought going home would be great…and some of the students were right, and some of the students were just, you know, making it up, just to be involved in what was going on.” [Assistant head S] “You can ask the child, you can ask the parents, but they don’t always necessarily know or they can’t remember, so yeah, I guess that for me, that was the main thing is really like, I think I, I guess I just had to accept that it was never going to be fully accurate.” [Teacher S]
	The use of more flexible digital seating plans, CCTV^d^, mobile proximity-tracking devices, and access cards could improve the accuracy of contact tracing, although concerns around privacy, acceptance, and technical limitations are prevalent among staff.	“Okay (in relation to CCTV), my personal opinion is it wouldn’t be ethical; I think it is the wrong way to use it…because you are literally spying on a person.” [Teacher S] “(in relation to proximity tracking) also I’m aware of the workforce needing to be in, and I don’t want false alerts that mean that we can’t run our school effectively because we’ve had to send people home, so some of that it needs to be utterly reliable.” [Assistant head S]
	No standardized procedures exist for storing and sharing information with authorities, and a digital system for interagency collaboration could assist schools’ contact-tracing efforts.	“Public Health England, actually the communication is dreadful. Our head teacher has had to sit on the phone spelling out her name, the school’s name…it’s all done in a really old-fashioned admin way.” [Assistant head S] “From first notification, we should just be able to upload all our information into a hub or a central record for our school.” [Assistant head S]
**Social distancing, contact-tracing provisions, and other COVID-19–related measures impact school functioning and place additional demands on staff and pupils.**		
	Measures have a negative impact on the teaching of practical subjects and other aspects of school life, although positive changes were also observed.	“The more practical subjects have been definitely limited just because the teachers have been going to the students rather than the usual other way around, yes.” [Assistant head S] “We’ve seen things like cross-year bullying completely vanish by keeping those students separated.” [Teacher S]
	Staff have taken on additional responsibilities and roles, and their workload has increased.	“We rewrote all of our schemes of learning to see if we could minimize the amount of physical contact the students had, while still being able to learn the skills we were trying to teach them.” [Teacher S] “Aa teacher, my role is [to] be sure that in the class, they’re wearing masks, be sure that they sanitize their hands, and they’re sitting where they’re supposed to sit. Be sure that I have windows and door open.” [Teacher S]
	The behavior of students, along with factors inside and outside of school, could further complicate efforts to improve contact-tracing and social distancing provisions.	“I think with technology, it’s all dependent on students having something, and yes, without…with our all good intent and purposes, sometimes it’s a battle trying to get students a pen [laugh] never even mind you know, something digital.” [Teacher S] “You know the…the breakages of equipment in school is…is…is unbelievable, I think, when you think about it…I don’t know. I…I can’t quite get my head round what that would look like.” [Assistant head S]

^a^S: secondary.

^b^P: primary.

^c^PE: physical exercise.

^d^CCTV: closed-circuit television.

#### Theme 1: Social Distancing Measures Were Put in Place to Prevent and Limit High-Risk Interactions; However, Blind Spots Exist

School staff in both primary and secondary schools were attempting to follow national guidance; however, there appeared to be significant differences regarding the provisions they had in place to manage social distancing. In primary schools, where learning required group work and interaction (especially in early years), rules around social distancing and strict seating arrangements in the classroom were not considered appropriate. When possible, however, staff would choose seating arrangements that would minimize face-to-face contact, such as rows or a horseshoe. Each class would be considered a “bubble,” whereby students would only mix with other students and staff in their bubble.

School staff in secondary schools used a number of additional systems and provisions that were in place. In the classrooms, strict seating arrangements were in place and participants reported using digital seating plans consistently; these had been generated by a digital system designed for classroom management. Bubbles were not used. During breaks, students would only mix with other students in their year group and there was not an expectation for students to socially distance within their year group. In the classroom, teachers would remain in a designated area 2 m apart from their students, and they continued to teach across different year groups.

To minimize interactions between different year groups in common areas, school staff in both primary and secondary schools implemented measures, such as staggered break times, keeping year groups separate, zoning, and 1-way systems. All staff were asked to keep at least 2 m distance from their colleagues, and changes were made to staff rooms to prevent close interactions.

Participants reported that high-risk interactions appeared to occur despite the measures that were put in place to prevent them. Staff members would not always keep their distance from their colleagues, and space limitations in some classrooms did not always allow them to stay 2 m apart from their students; and students in the same year groups were free to interact with each other in close proximity during break times in communal areas, such as halls and dining rooms. In secondary schools, science classes in particular presented challenges as there were instances when teachers where required to leave their designated area and approach the students to assist them with their experiments. One participant who was teaching science explained that it would be helpful to be reminded when they were in close proximity to their students. Receiving alerts from a digital system could potentially help prevent lengthy and risky interactions.

#### Theme 2: School Staff Use Both Manual and Digital Methods to Support Contact Tracing; However, Greater Accuracy Is Needed

In primary schools, where students and staff in each class formed a bubble, participants described contact tracing as a relatively simple process. When a positive case was identified, all students and staff in the same bubble were considered close contacts and were asked to isolate by the school. When students shared classes or activities with students from different bubbles, all those in the presence of a positive case would be sent into isolation. There was a perception among participants that primary school students could not be trusted to identify their close contacts. As a result, investigations often did not take place and isolating everyone in the same class or activity with the positive case was considered a far more practical option.

In secondary schools, large numbers of students were mixing across their year group in common areas. Teachers also taught across multiple year groups. As a result, contact tracing involved elaborate investigations. Digital seating plans were used routinely to identify close contacts in the classroom. Manual investigations, such as interviews with teachers, students, and parents, were also conducted by the school’s senior leadership team to identify social contacts. In 1 (16.7%) school, CCTV footage was reviewed routinely by staff to identify contacts in common school areas. Participants reported that maintaining the anonymity of the case was not a priority in contact-tracing investigations and stressed that no complaints had been raised by students, parents, or staff.

Participants, however, expressed strong concerns that manual investigations were not accurate. They admitted that seating plans were not always properly updated; participants would forget or struggle to update them if using alternative seating arrangements, and sometimes, students changed their allocated seat without first asking or informing their teacher. Participants explained that they would often keep separate files or instead take a quick photograph of the classroom. Recollection of close contacts by the positive cases was not always felt to be reliable, and participants would also come across contradictory accounts. Combined with the lack of any physical evidence of close interactions, participants had to manage these situations to the best of their ability and were relying heavily on their own memory and judgment.

Participants believed there was a need to improve the accuracy of contact tracing inside and outside the classroom and that digital solutions could help. Specifically, allowing students to update seating plans by scanning quick response (QR) codes on seats/tables was seen to have the potential to lead to more accurate plans and more flexible seating arrangements, along with more compliance from students. Participants felt CCTV cameras, which were already in place in all the schools, could help identify close contacts in areas such as dining halls. Mobile systems that could measure and track proximity between students and teachers (ie, proximity-tracking devices) were considered a valuable approach to improving accuracy. Less interactive technologies, including access cards, to monitor access in common school areas were also considered promising if they provided accurate information on the whereabouts of staff and students.

Despite their enthusiasm, participants did have reservations and concerns about these technological solutions. In relation to mobile proximity tracking, they were concerned about privacy violations as they felt that students would be monitored constantly within the school. They also highlighted the issue of consent and the need for everyone in the school to use this system. Furthermore, there were concerns about false proximity alerts that could send staff to self-isolation unnecessarily. Participants overwhelmingly expressed the view that manually reviewing CCTV footage would be time-consuming and therefore not feasible, and there were concerns regarding technical limitations of the CCTV system (eg, areas in the schools that are not covered by cameras, footage may not be clear and detailed enough to allow the identification of students). Furthermore, the majority of schools discussed by participants currently use CCTV footage only in exceptional circumstances to identify students who are involved in serious incidents, such as fights. Therefore, there were concerns among some participants about extending this to the infection control scenario as this would require frequent use of the system, which they considered invasive. Technical issues along the time required to review footage would make it difficult to make its use a common school practice.

Participants also highlighted challenges when contacting public health authorities (specifically the now disbanded Public Health England [PHE]) to report positive cases and to receive advice and support with contact tracing. The system required multiple phone calls and repeating the same information to different members of staff. Introducing a digital record that could be shared between schools and public health authorities was seen as a way to improve their communication and collaboration. However, this was not a challenge experienced by all participants, as the schools whose staff felt confident in managing contact tracing were not required to contact the authorities.

#### Theme 3: Social Distancing, Contact-Tracing Provisions, and Other COVID-19–Related Measures Impact School Functioning and Place Additional Demands on Staff and Pupils

Social distancing along with other provisions aiming to keep pupils and staff safe has had a profound impact on school life. Participants explained that the teaching of more practical subjects was especially difficult. In schools where zoning had been implemented, students in different year groups stayed in separate areas of the schools. As a result, access to rooms with equipment needed for drama, music, science classes, and outdoor facilities needed for physical exercise (PE) was restricted. Participants recounted trying to move equipment between classes but that this had significantly impacted the quality of teaching. PE sessions were also reduced in order to allow for cleaning of changing rooms.

To manage these changes, participants had taken on additional responsibilities, including moving equipment between classes, adjusting the curriculum, making sure to sterilize their own working areas, reminding students to wear masks and keep their distance, following up on students who tested positive, and managing the whole school response. Participants also highlighted that factors outside the school’s control, such as mixed messages on what constitutes close contact in the school, not making testing obligatory for students, and change of rules around mask wearing and temperature testing, had further complicated their efforts to make the school a safe environment for their staff and students.

Despite the challenges, participants observed positive changes in the school environment. Cross-year bullying stopped in 1 (16.7%) school since year groups were kept separate, while enhanced cleaning and insistence on hygiene measures led to a perceived reduction in outbreaks of other illnesses. Some participants also noted that their schools would permanently implement some of the changes.

## Discussion

### Principal Findings

#### Introducing CCTV, proximity tracking, and access cards into contact tracing could increase the accuracy and speed of contact tracing

This study explored the experiences of school staff in managing COVID-19, along with their requirements for digital solutions that can enhance their efforts to improve contact tracing and limit the spread of COVID-19 among students and staff. Although schools implemented government recommendations, school staff still found it difficult to limit and appropriately monitor high-risk contacts and strongly believed there was a need to improve accuracy in contact tracing. School staff in secondary schools faced more serious challenges as students interacted and congregated with other students in their year group, with year group sizes reaching 200, 300, or more students [55]. In primary schools, students were reported to have close contact with other students within their bubble, which includes an average of 30 students [56].

Findings suggest an opportunity for digital systems, particularly access cards, proximity tracking, and CCTV, to improve the accuracy and efficiency of contact tracing. Manual investigations (interviews with teachers, students, and parents) and reviews of seating plans were not considered accurate, as they relied on individuals’ ability to recall interactions and to keep their records up to date. Introducing automation in contact tracing could increase the speed and accuracy of investigations and potentially have a more profound effect in secondary schools. The contacts of secondary school students can grow exponentially in the time it takes to conduct manual investigations or if some of the close contacts are missed in this process. Delays in quarantining or testing close contacts allows more space for the virus to spread within school.

#### Need to Understand and Address Privacy Expectations and Concerns Among Various School Stakeholders Before Introducing New Tools

Introducing mobile proximity-tracking devices and new uses for CCTV (primarily tracking of individuals for contact-tracing purposes) were felt to be promising but raised privacy concerns among the staff. Participants were specifically worried that the introduction of these solutions would result in the constant monitoring of students within the school. To address these concerns, more conceptual work is needed to unpick the notion of privacy and we should try to understand what the privacy expectations are across different settings within the schools. Although there is no universal definition of privacy, the concept of “reasonable expectations of privacy” determines in which places and activities a person can expect to have a right to privacy [57]. The concept highlights that expectations of privacy largely depend on the setting and circumstances. To this end, we should ask ourselves, What may be the reasonable expectations of privacy within schools? Schools are required to hold personal information about students and their families [58], and they are expected to monitor and supervise behavior to support the welfare and education of their students [59]. Individuals can also have different expectations, depending on where they are within the schools. There is probably no expectation of privacy in the classroom, where attendance and performance are strictly monitored, while school toilets are a place where individuals are not expected to be observed.

This study explored the experiences and perceptions of teachers, but there may be different expectations of privacy among the various stakeholders, including different members of the school community (teachers, parents, students). Parents many expect the school to inform them about their child’s behavior, whereas students may not be keen for the school to observe or report on their behavior and keep their behavior private. Overall, digital solutions for contact tracing designed for schools should therefore prioritize security measures to address concerns around privacy. These measures, along with clear explanations regarding how they are going to be used and for what purposes, should be highlighted in the communication with the school community. Co-designing the solutions and exploring the concerns and expectations of privacy among different stakeholders will increase the likelihood that any solutions would be accepted with high uptake.

There were particular concerns about the technical requirements in using CCTV for contact-tracing purposes. Facial recognition technology, where captured images are compared against a database of pre-existing personally identifiable images in the system, should make it easier and faster to identify individuals [60]. However, this technology could be considered invasive, and its application has been met with resistance [25,29]. Exploring views regarding the application of facial recognition among members of the school community could provide more clarity on whether they would consider its use appropriate.

#### Contact-Tracing Tools Should Place Minimal Burden on the School, Staff, and Students

Participants were concerned about the demands of any contact-tracing systems on their schools’ budget. Buying, fixing, and replacing equipment were suggested to be extremely difficult for schools, and there was also concern that mobile proximity tracking could generate false alerts and send the staff unnecessarily into isolation. This can lead to staff shortages and the need to bring in temporary staff at a great cost for the school. Any digital solution designed for schools should come with available technical support as schools are unlikely to cope with the ongoing demands of maintaining such a system. This study further suggests that accuracy (including avoiding false positives, which would unnecessarily require students and staff to isolate) should be 1 of the system’s key features. Furthermore, reducing the need for involvement of staff and students in the application and use of any digital solution seems crucial. The staff have already taken on many responsibilities, and increasing their heavy workload would put additional pressure on them. Expecting students to take on an active role as users could create additional barriers, considering the different maturity levels among this population [61]. Therefore, digital solutions should be designed to fit in with schools’ workflow and routine and require minimal interaction from individuals.

### Limitations

This study concentrated on schools in the southwest of England. We chose this focused approach due to the lack of research in the area and the desire for an in-depth exploration of school staff’s experiences. To build on our findings, a larger UK-wide quantitative study could enable broader generalizability. The topic guide was not pilot-tested, as the pace at which the COVID-19 pandemic has been unfolding required the research team to provide results and insight at a fast pace, thus creating additional time constraints.

Although the study achieved purposive sampling at the school level, that was not possible at the participant level. This study included a relatively small numbers of senior school staff (managers and teachers), mainly due to the high workload experienced by schools coping with the impact of the pandemic. Since senior members are tasked with managing the whole school response, they could have provided more insights regarding the impact and applicability of digital solutions. We did not manage to recruit staff members such as reception staff, other administration staff, and cleaners, and these staff members could have provided valuable insights into COVID-19 management in schools.

### Conclusion

This qualitative study found that school staff reported a need for better COVID-19 mitigation measures, especially in secondary schools, and digital tools, such as CCTV and mobile proximity tracking and access cards, were described as potential solutions. It is important to ensure that any tools designed for schools prioritize privacy concerns and have minimal impact on staff, pupils, and day-to-day management. Further qualitative work would enable exploration of acceptability, feasibility, and engagement with the specific digital solutions that have emerged and explore the views of other key stakeholders, such as students, parents, and decision makers.

This study explored challenges faced by school staff in implementing COVID-19 measures and different provisions between secondary and primary to identify suitable digital tools. Overall, findings can help researchers and practitioners who work in different contexts and settings understand the particular challenges faced by school staff and inform further research on the design and application of digital solutions.

## Abbreviations

BAME: Black, Asian, and minority ethnic
CCTV: closed-circuit television
CoMMinS: COVID-19 Mapping and Mitigation in Schools
FSM: free school meals
PE: physical exercise
PHE: Public Health England
QR: quick response
